# Magmatic gas percolation through the old lava dome of El Misti volcano

**DOI:** 10.1007/s00445-017-1129-5

**Published:** 2017-05-24

**Authors:** Yves Moussallam, Nial Peters, Pablo Masias, Fredy Apaza, Talfan Barnie, C. Ian Schipper, Aaron Curtis, Giancarlo Tamburello, Alessandro Aiuppa, Philipson Bani, Gaetano Giudice, David Pieri, Ashley Gerard Davies, Clive Oppenheimer

**Affiliations:** 1 Department of Geography, University of Cambridge, Downing Place, Cambridge, CB2 3EN UK; 2 Observatorio Vulcanológico del Ingemmet (OVI), Arequipa, Peru; 3 Nordic Volcanological Center, Institute of Earth Sciences, Sturlugata 7 – Askja, 101, Reykjavik, Iceland; 4 School of Geography, Environment and Earth Sciences, Victoria University of Wellington, PO Box 600, Wellington, 6140 New Zealand; 5 Jet Propulsion Laboratory-California Institute of Technology, 4800 Oak Grove Drive, Pasadena, CA 91109 USA; 6 Istituto nazionale di geofisica e vulcanologia, sezione di Bologna, Bologna, Italy; 7 Dipartimento DiSTeM, Università di Palermo, Via archirafi 36, 90146 Palermo, Italy; 8 Istituto Nazionale di Geofisica e Vulcanologia, Sezione di Palermo Via La Malfa, 153, 90146 Palermo, Italy; 9 Université Clermont Auvergne, CNRS, IRD, OPGC, Laboratoire Magmas et Volcans, F-63000 Clermont-Ferrand, France

**Keywords:** Volcanic hazard, Arequipa, Outgassing, ASTER, Multi-GAS, Trail by fire

## Abstract

The proximity of the major city of Arequipa to El Misti has focused attention on the hazards posed by the active volcano. Since its last major eruption in the fifteenth century, El Misti has experienced a series of modest phreatic eruptions and fluctuating fumarolic activity. Here, we present the first measurements of the compositions of gas emitted from the lava dome in the summit crater. The gas composition is found to be fairly dry with a H_2_O/SO_2_ molar ratio of 32 ± 3, a CO_2_/SO_2_ molar ratio of 2.7 ± 0.2, a H_2_S/SO_2_ molar ratio of 0.23 ± 0.02 and a H_2_/SO_2_ molar ratio of 0.012 ± 0.002. This magmatic gas signature with minimal evidence of hydrothermal or wall rock interaction points to a shallow magma source that is efficiently outgassing through a permeable conduit and lava dome. Field and satellite observations show no evolution of the lava dome over the last decade, indicating sustained outgassing through an established fracture network. This stability could be disrupted if dome permeability were to be reduced by annealing or occlusion of outgassing pathways. Continued monitoring of gas composition and flux at El Misti will be essential to determine the evolution of hazard potential at this dangerous volcano.

## Introduction

Lava domes are the extruded part of a magma risen through a conduit. Typically associated with silicic (rhyolitic to andesitic) magmas, lava domes are often unstable and capable of generating pyroclastic density currents. Dome collapse events have been documented at volcanoes such as Soufrière Hills (Montserrat; e.g. Watts et al., 2002), Mount Unzen (Japan; e.g. Sato et al., 1992), Mount St. Helens (USA; e.g. Mellors et al., 1988) and Merapi (Indonesia; e.g. Komorowski et al., 2013). Critical in determining whether or not an otherwise gravitationally stable dome will become overpressured and collapse is the nature of magmatic outgassing through either the conduit wall or fracture networks (e.g. Jaupart and Allègre, 1991; Gonnermann and Manga, 2003; Boudon et al., 2015). While such considerations have been explored theoretically (e.g. Sparks, 1997; Melnik and Sparks, 1999, 2005; Hale and Mühlhaus, 2007) the inherent hazard associated with working on active lava domes has limited in situ collection of data on gas compositions, limiting data to that from remote sensing observations (e.g. Oppenheimer et al., 2002; Edmonds et al., 2003; Holland et al., 2011) with only a few direct measurements (e.g Soufrière Hills; Hammouya et al., 1998).

**Fig. 1:**
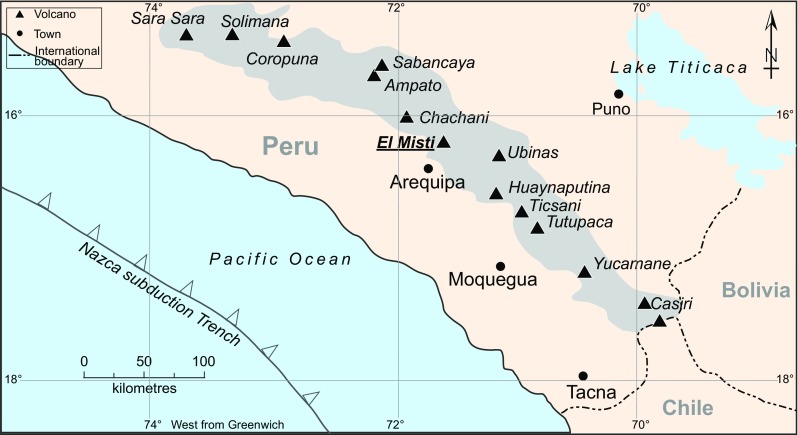
Location map showing the location of El Misti volcanoes together with the location of all Peruvian Holocene volcanoes and the Nazca subduction trench. The centre of Arequipa (one million inhabitants) is located 18 km from El Misti’s summit

**Fig. 2:**
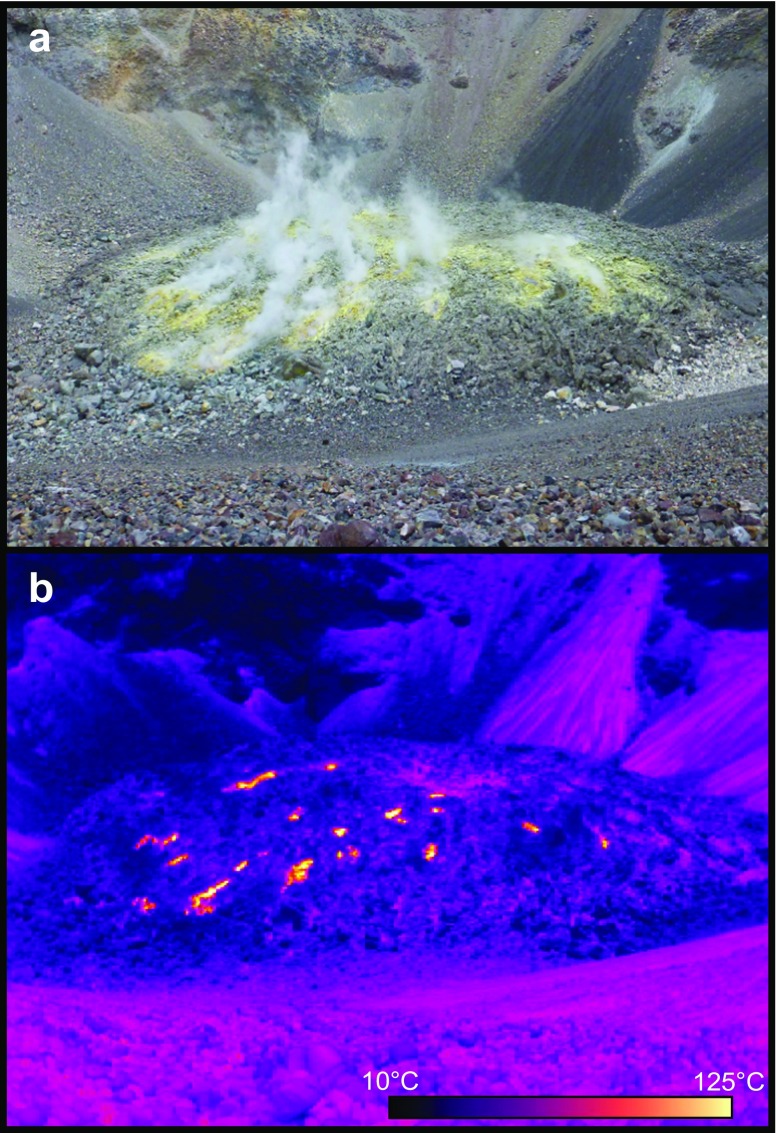
Visible (**a**) and infrared (**b**) images of the lava dome of El Misti taken on 1 December 2015 from within the crater, looking south. The dome is about 150 m across

El Misti volcano, southern Peru (Fig. 1), is close to Arequipa, the second largest city in the country, with about one million inhabitants. El Misti is a composite stratovolcano composed of four stratocones (Thouret et al., 2001). The youngest, Misti 4, forms the current summit of the volcano and has erupted at least ten times since 11,000 years. B.P producing pyroclastic-surges and lahars that have travelled up to 13 km from the vent (Thouret et al., 2001). The last plinian eruption occurred ca 2050 years ago and produced a widely dispersed pumice-fall deposit extending ≥25 km from the vent (Thouret et al., 1995, 2001; Harpel et al., 2011; Cobeñas et al., 2012). Since then, eruptive activity has been mild, with minor events in 655–865 A.D., 1304–1398 A.D. (Thouret et al., 2001) and in 1440–1470 A.D. (Murúa, 1946, 1987); episodes of increased fumarolic or seismic activity, and small phreatic eruptions (Barriga, 1951; Zamácola and Jaureguí, 1958; Hantke and Parodi, 1966; Chávez Chávez, 1992; Simkin and Siebert, 1994; Thouret et al., 2001). Within the crater of El Misti is an old lava dome of unknown age, potentially as old as the last magmatic eruption (fifteenth century), on which a fumarole field is located (Fig. 2). The dome is covered with sulphur sublimate previously characterised by Birnie and Hall, (1974). The current fumarolic activity has persisted since at least 1787 (Thouret et al., 2001), and a persistent thermal anomaly of ∼+6 K has been identified at the summit in Advanced Spaceboune Thermal Emission and Reflection Radiometer (ASTER; Yamaguchi et al., 1998) thermal infrared images from 2000 to 2010 (Jay et al., 2013). ASTER is on-board NASA’s *Terra* spacecraft.

Despite a number of studies emphasising the hazards and risks of future volcanic activity of El Misti (e.g. Sandri et al., 2014), very little work has focused on its ongoing outgassing. Here, we present the first compositional data for the gas plume emitted from the El Misti lava dome together with an analysis of photographic, thermal camera and satellite images. We evaluate the results in terms of outgassing behaviour and the implications for hazard evolution.

## Methods

### In situ gas measurements

Gas composition data were obtained using a portable Multi-GAS instrument (Shinohara, 2005) deployed directly inside the crater and on the dome of El Misti (S 16° 17′ 57.22″; W 71° 24′ 20.69″; 5600 m a.s.l.). The instrument incorporated SO_2_, H_2_S and H_2_ electrochemical sensors. The SO_2_ and H_2_ sensors have calibration range of 0–200 ppmv while the H_2_S sensor had a calibration range of 0–100 ppmv. An NDIR sensor was used for CO_2_ and calibrated for 0–10,000 ppmv with an accuracy ±2%. A relative humidity (R.H.) sensor (Galltec) was used to measure H_2_O, providing a measuring range of 0–100% R.H. with an accuracy of ±2%.

The conversion from relative humidity to water mixing ratio was made following Buck (1981) and using the following equation:

1 $$ {\mathrm{H}}_2\mathrm{O}=\frac{\left\{6.1121\times \left(1.0007+3.46\times {P}^{-6}\right)\times \exp \left[\frac{17.502\times T}{240.97+ T}\right]\times \frac{\mathrm{Rh}}{100}\times {10}^6\right\}}{P} $$

where H_2_O is the absolute water concentration in parts per million by volume, *T* is the temperature in degrees Celsius, Rh is the relative humidity in percent and *P* is the atmospheric pressure in millibars. The gas temperature used in this equation is measured in real time by the Multi-GAS, the pressure is also measured by the Multi-GAS and assumed to remain constant during the measurements. All sensors were housed inside a weatherproof box, with the ambient air sampled via Teflon tubing connected to a HEPA filter fed through an inlet in the box and circulated via a miniature 12-V rotary pump through the sensors. An on-board data-logger captured measurements at a rate of 1 Hz. The complete system is powered by a small (6 Ah) 12 V LiPo battery. Similar systems have now been successfully deployed at many volcanoes (e.g. Aiuppa et al. 2011, 2012, 2014, 2015, Moussallam et al. 2012, 2014, 2016). All sensors were calibrated in the laboratory at INGV Palermo (October 2015), with target gases of known concentration. The differences in response time for the different sensors were corrected by finding the lag times from correlation analysis of the various time series. Ambient air composition was subtracted from the CO_2_, H_2_O and H_2_ data, SO_2_ interference on H_2_S data was calibrated and corrected. Multi-GAS measurements were taken on 1 December 2015 for 90 min. Post processing was performed using the Ratiocalc software (Tamburello, 2015).

### Infrared and visible camera

Thermal infrared images were acquired from within the crater rim pointing at the dome. Images were taken using an OPTRIS PI400 camera (spectral range of 7.5–13 μm with an optical resolution of 382 × 288 pixels), fitted with an 8-mm lens providing a FOV of 62°×48°. Thermal images were acquired on 1 December 2015 using the temperature range setting of −20 to 100 °C.

Photographs of the lava dome were acquired from the crater rim by OVI personnel during repeated ascents in the period from 2007 to 2016.

### Satellite observations

We used land surface temperature maps derived from ASTER thermal images (AST08 Land Surface Temperature, or LST, product at 90 m/pixel spatial resolution), short wavelength infrared Advanced Land Imager (ALI) images (nine bands from 0.4 to 2.4 μm at 30 m/pixel spatial resolution, with a panchromatic band (PAN) at 10 m/pixel spatial resolution—Ungar et al. 2003), Hyperion hyperspectral data (196 usable bands from 0.4 to 2.4 μm at 30 m/pixel spatial resolution) (Pearlman et al. 2003) and high resolution visible images (from Google Earth) to track potential changes in activity at the lava dome in the 15 years leading up to the fieldwork presented here. Hyperion and ALI are on-board NASA’s *Earth Observing 1* (EO-1) spacecraft. Observations by *EO-1* were obtained via the NASA Volcano Sensor Web (VSW, e.g. Davies et al., 2015), which utilizes advanced spacecraft operations software developed to streamline the process to re-task spacecraft as quickly and efficiently as possible to effect rapid data acquisition of dynamic targets (Chien et al., 2005).

All ASTER granules covering El Misti were acquired from NASA Reverb at both level 1b (radiometrically and geometrically corrected at sensor radiance) and at level 3 AST08, extending the coverage of Jay et al., (2013) from 2010 to late 2016. The AST08 LST dataset is produced by applying the temperature emisivity seperation algorithm (TES) to atmospherically corrected images (Gillespie et al., 1998). We used DA White’s Aster Preprocessing Toolkit (APTK, White 2016) to extract the correct rational polynomial coefficients (RPCs) from the level 1b data, which were then used to orthorectify the LST product to the 30-m shuttle radar topography mission digital elevation model with bilinear resampling. All images were classified by inspection as either cloudy or cloud free, then pixels in the summit region were extracted from the cloud free images. Previous studies have manually selected thermal anomalies in ASTER images of Andean volcanoes (Jay et al. 2013); however in this study, we opted for an automated approach for expediency. Blank zero padding pixels that fell within the summit region were removed using a threshold and a simple measure of the thermal anomaly, *T*_anomaly_, was calculated:

$$ {T}_{\mathrm{anomaly}}={T}_{\max } - {T}_{50\%} $$

Where *T*_max_ is the maximum temperature in the summit area, and *T*_50%_ is the 50th percentile (or median) temperature. This gives a measure of the maximum temperature relative to a robust estimate of the surrounding temperature. We limited our study to night time images, when the contrast between cool ground and geothermally heated areas is highest. The LST maps have a resolution of 90 m and allowed us to track changes in temperature over a broad area of the summit of El Misti.

Time series images of the lava dome in the visible where taken from Google Earth from the period of 2002 to 2016 using images from Digital Globe, NASA, Landsat/Copernicus and CNES/Astrium.

Images from the ALI and Hyperion on EO-1 were acquired around the fieldwork period on 11th October and 4th December 2015. Additional ALI images from 2002, 2003 and 2014 were also downloaded from the USGS Earth Explorer archive. All ALI images were processed to level 1T (precision orthorectified product). The short wavelength infrared bands of ALI have a resolution of 30 m, and allow small but very hot features to be identified, however all images were acquired during daytime so small thermal anomalies might be lost amid reflected sunlight. The VSW processes all Hyperion data searching for thermal anomalies (see Davies et al., 2006) using software originally developed to do this thermal emission detection on-board the spacecraft (the Autonomous Sciencecraft Experiment (ASE)—Chien et al., 2005; Davies et al., 2006), and has proved capable of detecting small thermal anomalies even in daylight. The Hyperion pixel brightness temperature detection limits of the ASE thermal classifier software are 426 K at 2.28 μm and 530 K at 1.65 μm (Davies et al., 2006).

## Results

### Gas composition

**Fig. 3:**
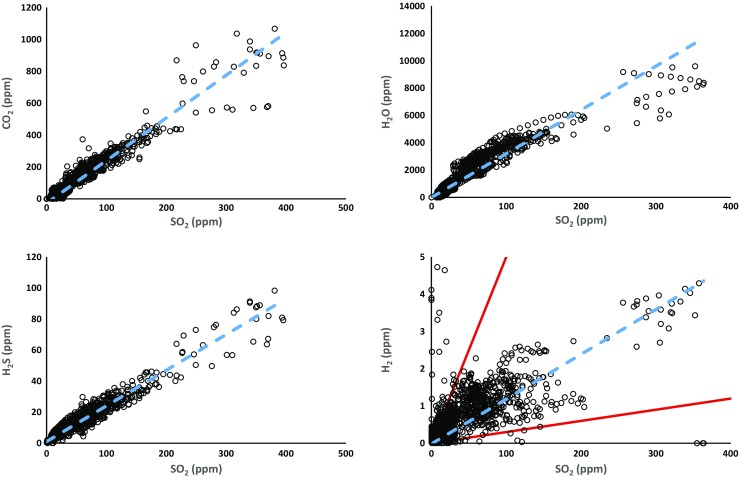
CO_2_, H_2_O, H_2_S and H_2_ vs SO_2_ scatter plots of the mixing ratios in the El Misti plume. Measurements were taken on 1 December 2015 for 30 min at an acquisition frequency of 1 Hz. Least-square regression lines are shown in dotted blue on each plot. Red lines on the H_2_ vs SO_2_ scatter plot show a conservative estimate of the range of potential gas ratio

We obtained 30 min of very high quality measurements presented in Fig. 3, which shows four scatter plots of SO_2_ vs CO_2_; H_2_O; H_2_S and H_2_ mixing ratios in the El Misti plume. H_2_O and CO_2_ concentrations and mixing ratios in the volcanic gas are shown after subtraction of their respective mean concentrations in ambient air (measured by the Multi-GAS in the crater but prior to entering the plume). H_2_S mixing ratios are shown after correction for laboratory-determined interference with SO_2_ gas (16%). The strong positive covariations observed between SO_2_ and the other detected volatiles confirm a single, common, volcanic origin. The gas/SO_2_ molar plume ratios were obtained by calculating the gradients of the best-fit regression lines. Scatter plots yield CO_2_/SO_2_ molar ratios of 2.7 ± 0.2, a H_2_O /SO_2_ molar ratios of 32 ± 3.4, a H_2_S/SO_2_ molar ratios of 0.23 ± 0.02 and a H_2_/SO_2_ molar ratios of 0.012 ± 0.002. Together these data yield molar proportions of H_2_O, CO_2_, SO_2_, H_2_S and H_2_ gases of 89, 7.5, 2.8, 0.6 and 0.03 mol% (Table 1). The correlation between H_2_ and SO_2_ mixing ratios is much weaker than for other species (*R* ^2^ value of 0.21 compared to *R* ^2^ values of 0.93, 0.88 and 0.94 for CO_2_, H_2_O and H_2_S vs SO_2_). The two red lines in Fig. 3 show a conservative estimate of the range of H_2_ to SO_2_ molar ratio from 0.05 to 0.003 that could be derived from the data.

**Table 1:** X/SO_2_ molar and mass ratios measured by multi-gas and gas composition of the mixed plume at El Misti volcano. Error are expressed as the standard error of the regression analysis and subsequent error propagation

Volcano	Gas	Mixed plume molar ratio (X/SO_2_)	Error (1σ)	Mixed plume mass ratio (X/SO_2_)	Error (1σ)	Mixed plume composition (mol%)	Error (1σ)
El Misti	H_2_O	32	3	9	1	89	9
	CO_2_	2.7	0.2	1.9	0.1	7.5	0.6
	SO_2_	1	0	1	1	2.8	0
	H_2_S	0.23	0.02	0.12	0.01	0.64	0.06
	H_2_	0.012	0.002	0.00038	0.00005	0.033	0.005

### Vent temperature

Parodi (1966) estimated the fumarole gas temperatures at over 250 °C (unknown date) stating that sulphur appeared melted in the sources’ emission gaps. Birnie and Hall (1974) estimated fumaroles temperatures of 100–125 °C while Thouret et al. (2001) indicate a maximum temperature of 220 °C measured in December 1997. In 2013 OVI personnel measured fumaroles temperatures between 270 and 310 °C. In 2015, our thermal camera measurements indicate a temperature in excess of 125 °C (temperature at which the image saturated). Given the distance at which the image was acquired and the small size of the vents, fumarole temperatures in excess of 200 °C are expected. Inspection of short wave infrared images from the ALI on EO-1 acquired in 2002, 2003, 2014 and 2015 reveals the presence of a small plume above the dome, but no thermal anomaly. This observation, together with the absence of incandescence observed on the dome at night, suggests vent temperatures below around 600 °C (basaltic rock is known to glow red at ∼700 °C; Decker and Christiansen, 1984).

### Time series observations

**Fig. 4:**
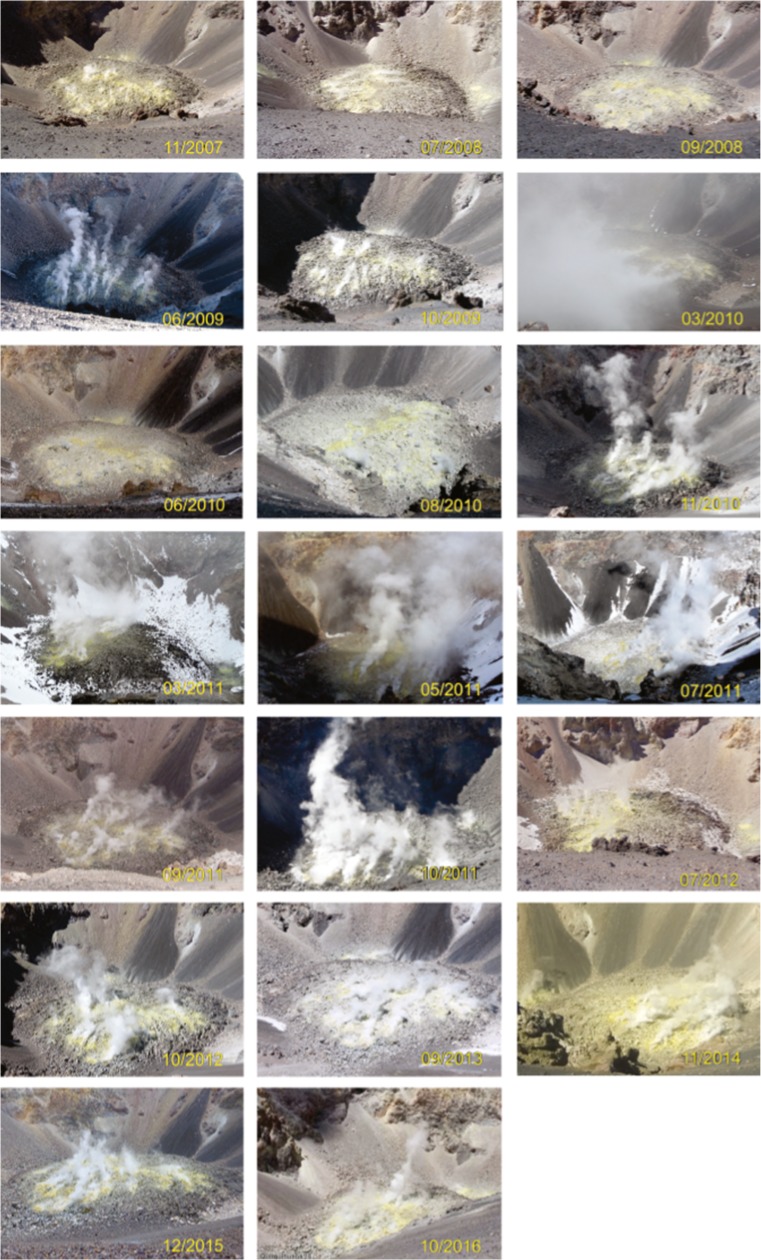
Still photograph of the El Misti lava dome taken from the crater rim during repeated ascent from 2007 to 2016 by OVI personnel. The pictures from 2008 were taken by Victor Aguilar (Universidad Naciona de San Agustin). Note the lack of changes in both the dome morphology and location of fumaroles

**Fig. 5:**
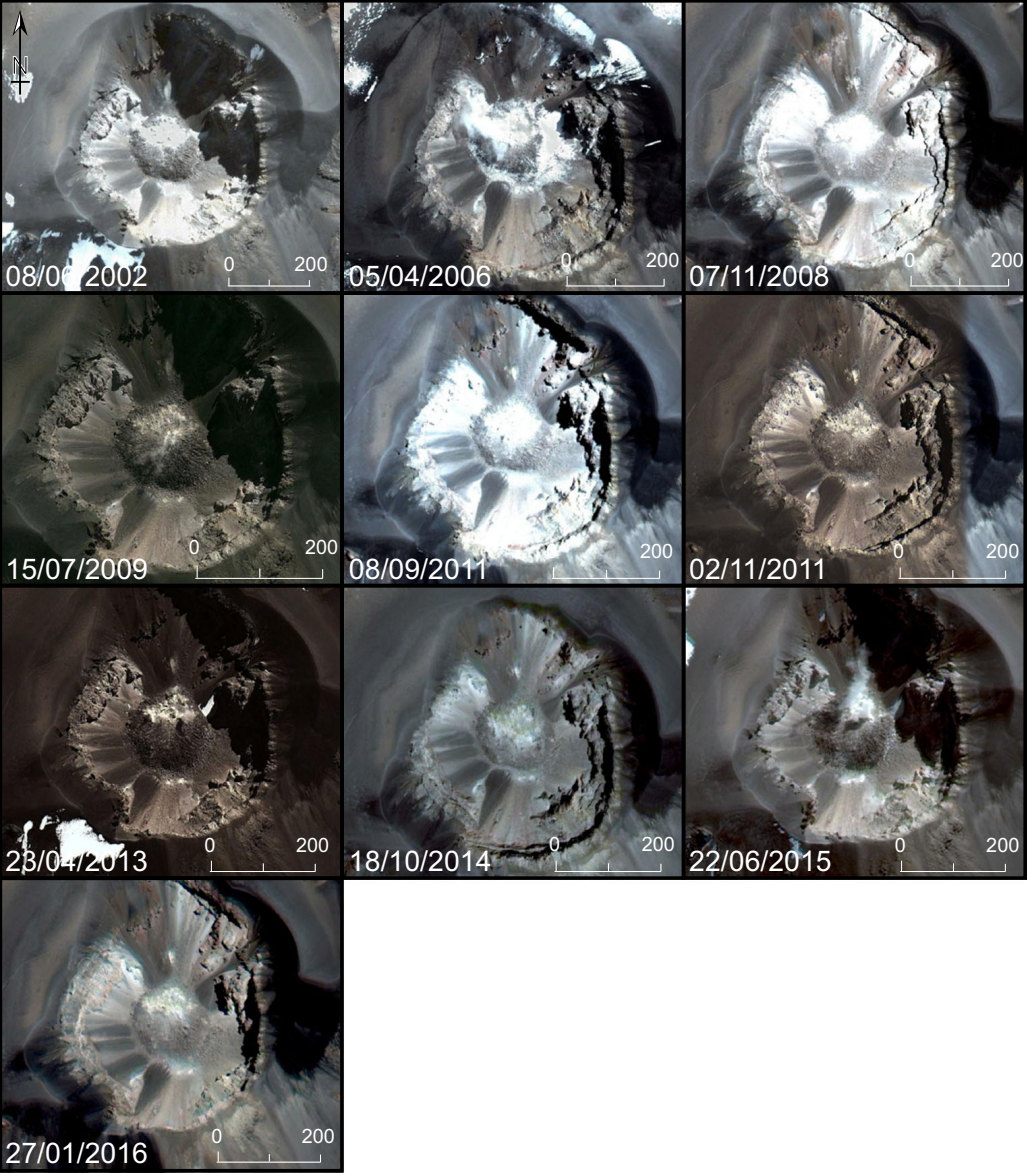
Satellite images of the El Misti lava dome from 2002 to 2016 taken from Google Earth and using images from Digital Globe, NASA, Landsat/Copernicus and CNES/Astrium. Note the lack of changes in both the dome morphology and location of the fumaroles field. Up is north on all images. The lava dome at the centre of each image is approximately 200 m in diameter

**Fig. 6:**
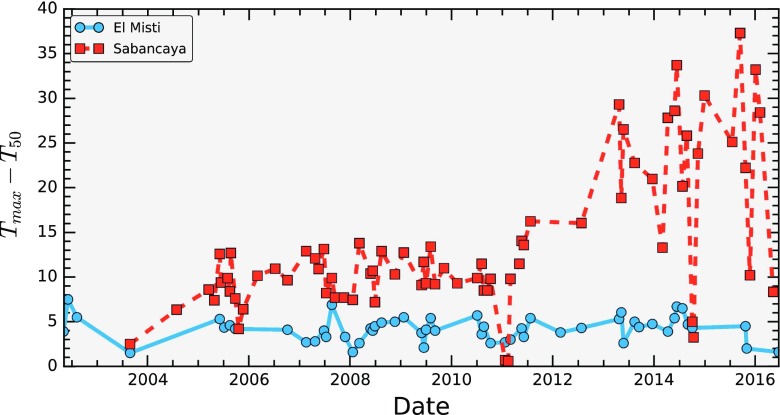
Time series of temperature anomalies (maximum temperature minus median temperature for all pixels in the summit area) derived from the ASTER AST08 land surface temperature product for El Misti and Sabancaya

Direct images of the dome taken from the crater rim from the period 2007 to 2016 are shown in Fig. 4. To our knowledge, the oldest published photograph of the dome dates from 1967 and is presented in Birnie and Hall, (1974). During the investigated period and since 1967, no changes in the dome morphology are apparent. The location of the fumarole field does not change, and while variations in the intensity of the outgassing can be seen, these may simply indicate variability in atmospheric conditions (e.g. variations in temperature- and humidity-dependent steam condensation) rather than true variability in gas flux. Periods of increased gas flux have, however, been documented at El Misti, such as in 2011 when a constant gas plume could be seen from Arequipa (OVI observations). Satellite observations at visible wavelengths spanning the period 2002 to 2016 are shown in Fig. 5. Consistent with field observations, these also show no variations in the dome morphology, area or situation of the fumarole field. ASTER observations of land surface temperature covering the period 2002 to 2016 are shown in Fig. 6. Compared with the time series from neighbouring Sabancaya volcano, which has shown varying levels of activity during the same period, the heat output from El Misti’s lava dome has stayed relatively constant, consistent with a ‘steady state’ outgassing and heat output. Inspection of short wave infrared images from the ALI on EO-1 acquired in 2002, 2003, 2014 and 2015 reveal the presence of a small plume above the dome, but no detectable thermal anomaly. Neither was any anomalous thermal emission detected in Hyperion images.

## Discussion

### A magmatic gas signature

The composition of the gases emitted from the El Misti lava dome is indicative of derivation directly from outgassing magma. This is apparent in the high SO_2_ concentration and the relatively low H_2_O/SO_2_ ratio, which together imply small to negligible contributions from a hydrothermal system. Given that we measured both H_2_O-H_2_ and SO_2_-H_2_S redox couples, the gas-melt equilibrium temperature and oxygen fugacity can be calculated. Following Giggenbach (1987, 1996) and using the thermodynamic data of Stull et al. (1969) provides two equations with two unknown:

2 $$ \log \frac{{\mathrm{H}}_2}{{\mathrm{H}}_2\mathrm{O}}=-\frac{12707}{T}+2.548-\frac{1}{2}\mathrm{logf}{\mathrm{O}}_2 $$

and

3 $$ \log \frac{{\mathrm{SO}}_2}{{\mathrm{H}}_2\mathrm{S}}=\frac{27377}{T}-3.986+\frac{3}{2}\mathrm{logf}{\mathrm{O}}_2-\mathrm{logf}{\mathrm{H}}_2\mathrm{O} $$

Solution yields an equilibrium temperature of 532 °C and a logfO_2_ equivalent to ΔQFM = +2.8 (where QFM refers to the quartz-fayalite-magnetite buffer, and where ΔQFM = logfO_2_−logfO_2_ of QFM at corresponding temperature) or ΔNNO = +2.0 (where NNO refers to the nickel-nickel oxide buffer, and where ΔNNO = logfO_2_ −logfO_2_ of NNO at corresponding temperature). Error on the measured gas ratios, especially on the H_2_/SO_2_ ratio results in non-symmetrical uncertainty of −96 and +133 °C on the equilibrium temperature and of −1.2 and +1.2 log units on the deviation from the QFM or NNO buffer. Equations for the QFM and NNO buffer used here are from Frost, (1991). The value of *f*H_2_O used here is 0.88 given that at 1 bar the fugacity of a gas is equal to its partial pressure and that P(H_2_O) = (Ptot × nH_2_O)/ntot = [(1 bar)(0.88ntot)]/(ntot) = 0.88 bar.

The equilibrium temperature of 532 °C is higher than the temperature at which scrubbing of magmatic gases by hydrothermal systems is expected to become significant (Symonds et al., 2001; Gerlach et al., 2008) giving confidence that the reported gas composition has not been affected by secondary processes other than cooling. The high oxidation state preserved by the gases further indicates limited interaction with low temperature rock (Giggenbach, 1987). The absence of contamination of the dome gas composition by hydrothermal fluids is consistent with the idea of Finizola et al., (2004) who suggested that the hydrothermal system at El Misti is sealed by hydrothermal alteration. The clear magmatic signature and high equilibrium temperature of the emitted gases, together with their high exit temperature (270–310 °C), suggest a relatively shallow magmatic source (see model from Stevenson, 1993). While the current composition of the magmatic source is unknown it may be similar to either the rhyolitic or andesitic magmas that interacted during the ca 2050 BP eruption (Tepley et al., 2013). Tepley et al. (2013) estimated the temperature of the andesitic magma at 940 ± 40 °C using pyroxenes pairs thermometry and the temperature of the rhyolitic magma at 816 ± 30 °C using groundmass ilmenite and magnetite thermometry. The temperature recorded by the gas composition (Eqs. 2–3) represent the temperature at which the gases were last in equilibrium and hence falls between the temperature of the vents and that of the magma.

### A permeable conduit and stable outgassing pathway

**Fig. 7:**
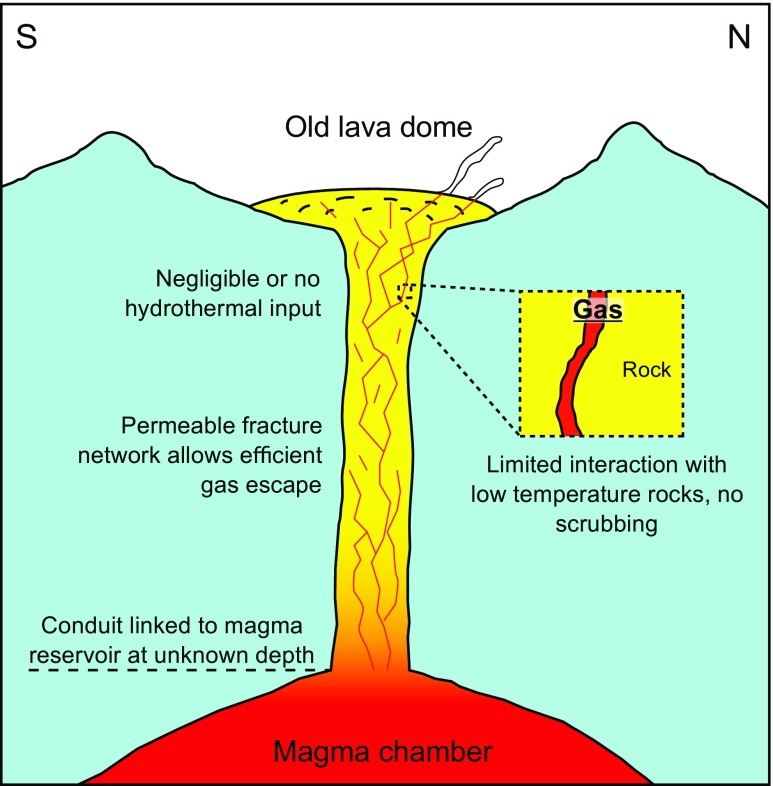
Schematic cross-section through the El Misti conduit highlighting the main conclusions from this study. Magmatic gases are released from a reservoir at unknown depth and quickly migrating to the surface through a network of established fracture with the conduit and lava dome. During ascent, the gas has very limited chemical interaction with the host rock and remains isolated from contamination by the surrounding hydrothermal system

Both direct (Figs. 2 and 4) and satellite (Figs. 5 and 6) observations show a very stable dome structure with little to no variations in terms of geometry, distribution of fumaroles, or heat output over at least the last 15 years. We note that previous InSAR surveys at El Misti also found no deformation of the edifice between 1992 and 2002 (Pritchard and Simons, 2004) nor between 2006 and 2009 (Gonzales, 2009). Together these observations imply a stable structure with established percolation pathways for the gas and little to no build-up of pressure within the edifice (Fig. 7). Figure 2 shows a strong correspondence between the location of thermal hotpots on the dome and the location of gas discharge.

While the exact date at which the lava dome formed within the crater is unknown, reports of fumarolic activity date back to the last magmatic eruption in 1440–1470 A.D. (Murúa, 1946, 1987). Several periods of increased fumarolic activity have since been reported (Thouret et al., 2001 and references therein) but no major eruptions have occurred, and it can hence be assumed that the current lava dome dates from the fifteenth century. The high equilibrium temperature we derived from the gas composition may partly explain the long-term preservation of permeable pathway through the conduit as the gas flow maintains a temperature too high for extensive precipitation of solids from the gas phase. Another possibility is that the relatively dry gas composition (unaffected by the hydrothermal system) is not prone to mineral precipitation before extensive cooling. The flux of gas coming out of the dome is fairly low (see discussion below) and it would hence be surprising if the gas pressure was responsible for maintaining a permeable fracture network.

### Implications for hazards and volcanic monitoring

The current ‘stable’ activity suggests an opportunity in terms of monitoring and hazard assessment in the sense that the gas composition, being largely unaffected by hydrothermal and scrubbing processes, would quickly respond to any changes happening in the magmatic system or conduit. For instance, a change of the temperature or composition of the magma following a recharge event—such as inferred for the last Plinian eruption in ca 2050 BC (Tepley et al., 2013)—should produce a measurable change in the equilibrium temperature and oxidation state of the gases. A change in the established outgassing pathways by fracture healing (e.g. Heap et al., 2015) or pore network collapse (e.g. Kennedy et al., 2016), for instance, could result in a decrease of the total gas flux. Visual observations (Fig. 4) point toward a very low SO_2_ flux at present, probably <50 t/day, rendering measurements by UV-based SO_2_ flux measurements challenging but maybe not impossible.

The report of phreatic eruptions since the fifteenth century (11 possible events, occurring in 1542?, 1599?, 2 May 1677, 9 July 1784, 28 July and 10 October 1787, August 1826?, August 1830 and 1831?, September 1869, and March 1870? see Parodi, 1966; Simkin and Siebert, 1994 and references therein) begs the question of their origin. The measured gas composition indicates that the current outgassing is isolated from the surrounding hydrothermal system. One hypothesis would be that following periods of increased precipitation, groundwater could interact with the hot magmatic gases generating small eruptions. Confirmed events do not however seem to occur preferentially in the rainy season. In the case of El Misti another hypothesis is that most recorded phreatic eruptions rather refer to periods of increased magmatic gas release either following a transient sealing then opening of the outgassing pathway or an increased flux of magmatic gas from depth. Distinguishing the relative roles of groundwater and magmatic fluids based on these historical observations recorded from a distance is not possible, and neither hypothesis can be dismissed. Both scenarios however would follow a perturbation of the system that has not been seen over our observational period.

## Conclusion

We measured the composition of gases emitted by El Misti in December 2015 and examined the evolution of the lava dome using satellite and direct observations dating back to 2002. The gas composition is indicative of magmatic outgassing with negligible contamination from a hydrothermal system. Together with the apparent stability of the lava dome and fumarolic field through time this implies the efficient release of magmatic gases from the reservoir to the surface through an established fracture network. Future gas monitoring campaigns will be worthwhile in order to track any potential evolution of the magmatic system and conduit.
